# Predictive factors for switching in patients with psoriatic arthritis undergoing anti-TNFα, anti-IL12/23, or anti-IL17 drugs: a 15-year monocentric real-life study

**DOI:** 10.1007/s10067-021-05799-0

**Published:** 2021-06-16

**Authors:** Mariagrazia Lorenzin, Augusta Ortolan, Giacomo Cozzi, Antonia Calligaro, Maria Favaro, Teresa Del Ross, Andrea Doria, Roberta Ramonda

**Affiliations:** Rheumatology Unit, Department of Medicine DIMED, Padova University Hospital, Giustiniani 2, 35128 Padua, Italy

**Keywords:** Biological therapy, Disease activity, Interleukins, Psoriatic arthritis, Survival analysis

## Abstract

**Objectives:**

We aimed to evaluate the (a) potential predictors of first biological disease-modifying anti-rheumatic drug (bDMARD) failure and (b) factors associated with failure of multiple therapies in psoriatic arthritis (PsA).

**Materials and methods:**

We enrolled consecutive PsA patients attending our unit and undergoing bDMARDs during 2004–2020. Disease characteristics, previous/ongoing treatments, comorbidities, and follow-up duration were recorded. Disease activity and functional and clinimetric scores were recorded at baseline and yearly and were compared between switchers and non-switchers, and within switchers according to the reasons for switching. Effectiveness was evaluated over time with descriptive statistics; multivariate Cox and logistic regression models were used to evaluate predictors of response and failure of multiple bDMARDs. Kaplan–Meier curves were used to assess differences in time-to-first bDMARD discontinuation. Infections and adverse events were recorded.

**Results:**

Two hundred sixty-four patients were included (117 (44.32%) females, mean age 56 years, mean PsA duration 15 years); 117 (44.32%) switched bDMARDs at least once. Switchers were mostly females, with higher Psoriasis Area and Severity Index and worse Health Assessment Questionnaire at baseline. Mean time-to-first bDMARD discontinuation was 72 months; 2-year and 5-year retention rates were 75% and 60%, respectively. Survival curves for anti-TNFα/anti-IL12/23/anti-IL17 were similar (*p* = 0.66). Main reasons for switching were inefficacy (67.52%) and adverse events (25.7%). Female sex was associated with a higher risk of first bDMARD discontinuation (HR = 2.39; 95% CI: 1.50–3.81) and failure of multiple bDMARDs (OR = 1.99; 95% CI: 1.07–3.69); initiating therapy before 2015 was protective (HR = 0.40; 95% CI: 0.22–0.73).

**Conclusions:**

Survival rate was good for anti-TNFα and other bDMARDs. Female sex was a predictor of first bDMARD discontinuation, unlike mechanism of action, comorbidities, and BMI.

**Table:** 

**Key Points** *• Drug survival in PsA patients was confirmed be greater for the first bDMARD administered.* *• In case of failure of the first bDMARD, switching/swapping proved a good treatment option, as reflected by a persistent satisfactory effectiveness with second-line bDMARDs and so subsequent switches.* *• Female sex may constitute a predisposing risk factor for flare and therapeutic switches.* *• Discontinuation or switching of biologics due to mechanism of action, comorbidities tolerability and BMI did not seem to impact first bDMARD withdrawal.*

**Supplementary Information:**

The online version contains supplementary material available at 10.1007/s10067-021-05799-0.

## Introduction

Psoriatic arthritis (PsA) is a chronic inflammatory disease characterized by articular and skin involvement [1]. Enthesitis, dactylitis, spine involvement, and extra-articular manifestations (e.g., uveitis, inflammatory bowel disease, and other comorbidities such as metabolic disorders and cardiovascular diseases) are typical occurrences in PsA [1–6]. The reported prevalence of PsA in the general population is about 1%, and the disease affects up to 30–40% (range 6–42%) of patients with psoriasis [7]. The disease often causes substantial functional impairment and decreased quality of life, if not diagnosed early and treated appropriately [8]. Non-steroidal anti-inflammatory drugs (NSAIDs) are commonly used to reduce the symptoms. Conventional synthetic disease-modifying anti-rheumatic drugs (csDMARDs) are recommended to treat the peripheral manifestations of the disease, thus improving clinical response and slowing disease progression. The European League Against Rheumatism (EULAR), the Group for Research and Assessment of Psoriasis and Psoriatic Arthritis (GRAPPA), and the Italian Society of Rheumatology (SIR) guidelines suggest to treat non-responsive patients with csDMARDs and those with an aggressive form with biological disease-modifying anti-rheumatic drugs (bDMARDs) [9–11]. Until the 2000s, PsA patients were treated with traditional csDMARDs, often with unsatisfactory results as it relates to disease control and radiographic progression [8]. The advent of biologics has changed the natural history of PsA by significantly improving quality of life and reducing damage progression. Biologics are currently recommended for the treatment of PsA in patients who respond inadequately to first-line treatment with NSAIDs and/or csDMARDs [9–11]. Randomized, placebo-controlled trials (RCTs) on currently available anti-tumor necrosis factor (TNF) α (infliximab, etanercept, adalimumab, certolizumab pegol, golimumab, biosimilars) agents have shown clinical improvement in the majority of PsA patients [12–18]. However, around 30% of PsA patients fail to respond to the first anti-TNFα and others experience adverse events, hence the question of whether anti-TNFα switching may be clinically beneficial [12–18]. Although anti-TNFα biologics are central to bDMARD treatment recommendations for PsA, new therapeutic alternatives have been approved, namely interleukin (IL)12/23 inhibitors (e.g., ustekinumab) and IL17 inhibitors (e.g., secukinumab, ixekizumab) as well as targeted synthetic disease-modifying anti-rheumatic drugs such as phosphodiesterase 4 inhibitor (apremilast) and Janus-activating kinase (Jak) inhibitor (tofacitinib) [19–23]. Notably, abatacept (cytotoxic T lymphocyte–associated antigen-4 immunoglobulin (CTLA-4-Ig)) has also been effective in patients with PsA [24]. bDMARDs have shown good tolerability and efficacy owing to a very high selectivity of therapeutic targets, a major step forward in PsA treatment [25].

However, according to the nationwide registries of drug continuation rate such as BIOBADASER (Spanish Registry of Adverse Events of Biological Therapies in Rheumatic Diseases), BSRBR (the British Society for Rheumatology Biologics Register), DANBIO (Danish Database for Biological Therapies in Rheumatology), and NOR-DMARD (Norwegian DMARD Registry), the treatment rate is considerable, with drug survival in the 63–82% range [26–30].

Our monocentric study evaluated PsA patients followed at the Rheumatology Unit of Padova University Hospital and undergoing long-term treatment with bDMARDs targeting TNFα (infliximab, etanercept, adalimumab, certolizumab pegol, golimumab), IL12/23 (ustekinumab), and IL17 (secukinumab), which have been approved for PsA treatment, in this chronological order. The aims of our study were to evaluate the (i) predictors of first bDMARD failure, including their mechanism of action; (ii) factors associated with failure of multiple therapies, including the frequency and the reasons for switching/swapping; and (iii) retention rate for anti-TNFα ustekinumab and secukinumab.

## Materials and methods

### Study population

This retrospective cohort study was conducted in a single tertiary center, Spondyloarthritis (SpA) Clinic of the Rheumatology Unit, Department of Medicine DIMED of Padova University Hospital (Italy). Consecutive patients classified as PsA according to CASPAR criteria [31] and initiating treatment with bDMARDs for a moderate or severe disease according to the EULAR/GRAPPA/SIR guidelines [9–11], during the period 2004–2020, were eligible. The bDMARDs investigated were infliximab, etanercept, adalimumab, certolizumab pegol, golimumab, ustekinumab, and secukinumab. Approval for the study was obtained from our institution’s ethics committee (n. 52,723), and all participants provided informed consent according to the principles of the Declaration of Helsinki. The dose and administration intervals of each bDMARD were predetermined in accordance with the manufacturer’s instructions. The dose or frequency of bDMARDs was not escalated arbitrarily. Infliximab was infused at 3 mg/kg at weeks 0, 2, and 6 and every 8 weeks thereafter. Depending on efficacy, patients would then receive gradual increments of 100 mg up to a maximum of 400 mg administered at 4- to 8-week intervals. The average dosage after 6 months was about 4.5 mg/kg every 8 weeks. Etanercept was administered twice weekly with an initial 25-mg subcutaneous dosage, often followed by 50 mg once weekly. Adalimumab was administered as a 40-mg subcutaneous dose every other week. Golimumab was administered by subcutaneous injection, 50 mg once every fourth week. Certolizumab was administered 400 mg subcutaneously, initially at weeks 2 and 4, followed by 200 mg every 2 weeks. Ustekinumab was administered as a 45-mg or 90-mg dose—according to body mass index (BMI)—at baseline, at 4 weeks, and every 12 weeks. Secukinumab was administered subcutaneously at a dosage of 150 mg or 300 mg as needed—according to the decision of the treating rheumatologist and the national registration indications of the drug—for severe psoriasis or multi-drug failure at weeks 0, 1, 2, 3, and 4 and every 4 weeks thereafter.

### Clinical and laboratory evaluation

Demographic, clinical, and laboratory data were collected at the time of bDMARD initiation and yearly, including the number of swollen and painful joints (swollen joint count (SJC), tender joint count (TJC)) out of 66/68 number of joints according to the American College of Rheumatology (ACR), Leeds Enthesitis Index (LEI), Psoriasis Area and Severity Index (PASI), visual analogue scale (VAS) pain and global health, C-reactive protein (CRP), erythrocyte sedimentation rate (ESR), Health Assessment Questionnaire (HAQ), and Disease Activity in PSoriatic Arthritis (DAPSA) [32]. Medical records were reviewed for information on patient age, disease duration, family history, smoking status, BMI, concomitant medications, and comorbidities (measured as Charlson Comorbidity Index [33]). Baseline characteristics were compared between switchers (≥ 1 switch/swap) and non-switchers. Switchers were patients who switched from the first prescribed bDMARD to one of the aforementioned bDMARDs. Non-switchers were patients who continued the first bDMARD. The physician decided the eligibility of an individual to switch biological medication. The duration of biological therapy, date of, and reasons (inefficacy, adverse events, infections) for switching/swapping were recorded.

### Statistical analysis

Data processing and statistical analyses were performed using SPSS, version 21.0 (SPSS, Chicago, IL, USA). Continuous variables are presented as medians and interquartile range and were compared using the Mann–Whitney *U* test. Categorical variables were analyzed using a chi-square test or Fisher’s exact test. Kaplan–Meier curves were used to assess differences in time-to-first bDMARD discontinuation according to the targeted cytokine. Survival of the second-line biological therapy was compared between swap and switch by log-rank test. Moreover, a Cox regression model with survival of the second-line biological therapy as outcome was built, in order to adjust for the effect of switch/swap as reason of the therapeutic change (loss of effectiveness/primary ineffectiveness/other reasons). A multivariable Cox proportional-hazard (PH) model was built to evaluate the influence of mechanism of action and negative prognostic factors for drug response on time-to-first bDMARD discontinuation. The following covariates were examined in this first model: drug mechanism of action (anti-TNFα/anti-IL12/23/anti-IL17), age, female sex, baseline comorbidities (measured by Charlson Comorbidity Index), BMI, baseline PASI, baseline HAQ, baseline DAPSA, polyarticular arthritis, and bDMARD initiation before 2015 (time frame reflects the unavailability of biological drugs with different mechanisms of action). Furthermore, a multivariable logistic regression model was built to assess the association between negative prognostic factors for drug response (independent variables) and failure of multiple bDMARDs (“multi-failure,” outcome). In this second model, the following covariates were examined: mechanism of action (anti-TNFα or anti-IL12/23 or anti-IL17), age, sex, and bDMARD initiation before 2015. *p* values ≤ 0.05 were considered significant.

## Results

Our study included 264 patients, 117 (44.32%) females, mean age of 56 (46–65) years, and mean PsA duration of 15 (10–22) years. One hundred forty-seven (55.68%) patients were non-switchers, and 117 (44.32%) were switchers. Switchers were mostly females (*p* < 0.001), with a lower PASI (*p* = 0.047), a higher prevalence of polyarticular arthritis (*p* = 0.048), and worse HAQ (*p* = 0.046) at baseline vs. non-switchers (Table 1). Mean time-to-first bDMARD discontinuation was 72 ± 58 months. Among 117 switchers, 54 (46.15%) patients underwent only one bDMARD switch, while 63 (53.84%) patients underwent ≥ 2 switches.

**Table 1:** Baseline characteristics of the monocentric cohort at the start of bDMARD therapy, comparison between switchers and non-switchers

Characteristics	Non-switchers (*N* = 147)	Switchers (*N* = 117)	*p* value
Female sex	**48 (32.65%)**	**69 (58.97%)**	** < 0.0001**
Age (years)	56.0 (46.0–65.0)	57.0 (49.0–65.0)	0.06
Psoriatic arthritis duration (years)	15.0 (10.0–22.0)	15 (10.0–21.0)	0.94
Polyarticular arthritis	**35 (23.81%)**	**42 (35.89%)**	**0.048**
Mono/oligoarticular arthritis	61 (41.50%)	54 (46.15%)	0.82
Axial involvement	42 (28.57%)	38 (32.48%)	0.95
Psoriasis duration (years)	25.0 (17.0–36.0)	24.0 (15.0–34.0)	0.67
Family history	9 (6.12%)	14 (11.96%)	0.07
Psoriasis	127 (86.39%)	102 (87.18%)	0.73
Inflammatory bowel disease	1 (0.68%)	3 (2.56%)	0.22
Uveitis	4 (2.72%)	0 (0%)	0.11
Tender joints (66/68 joint count)	5.0 (2.0–8.0)	4.0 (2.0–8.0)	0.44
Swollen joints (66/68 joint count)	2.0 (0.0–4.0)	2.5 (0.0–6.0)	0.60
VAS pain 0–10	7.0 (5.3–8.0)	7.0 (5.0–7.6)	0.90
VAS global health 0–10	6.0 (4.5–7.0)	6.5 (5.0–8.0)	0.35
CRP (mg/L)	6.0 (3.0–15.5)	5.5 (2.9–12.0)	0.37
ESR (mm/h)	17.0 (8.0–34.0)	19.0 (9.0–36.0)	0.42
DAPSA	20.2 (15.1–27.9)	18.9 (15.3–25.7)	0.67
Leeds Enthesitis Index (0–6)	0.0 (0.0–1.0)	0.0 (0.0–1.0)	0.69
Dactylitis (presence/absence)	18 (12.24%)	15 (12.85%)	0.23
HAQ	**0.5 (0.25–1.0)**	**0.62 (0.25–1.5)**	**0.046**
PASI 0–72	**1.0 (0.3–3.0)**	**1 (0.0–2.7)**	**0.047**
Smoking			
Non-smokers	91 (61.90%)	83 (70.94%)	0.46
Ever smokers	56 (38.10%)	34 (29.06%)	
BMI	25.3 (22.9–27.7)	25.4 (23.4–27.6)	0.86
Updated Charlson Comorbidity Index	1 (0–3)	1 (0–7)	0.79
Association therapy with a csDMARDs	56 (38.10%)	39 (33.33%)	0.61
First-line biological drug			
Anti-TNFα	120 (81.63%)	106 (90.59%)	0.75
Ustekinumab	15 (10.20%)	8 (6.84%)	0.88
Secukinumab	12 (8.16%)	3 (2.56%)	0.68

Significant results are highlighted in bold. Categorical variables are shown as number (%). Continuous variables are shown as medians and interquartile range. *p* ≤ 0.05 (between non-switchers vs. switchers)

*VAS* visual analogue scale, *CRP* C-reactive protein, *ESR* erythrocyte sedimentation rate, *DAPSA* Disease Activity in PSoriatic Arthritis, *HAQ* Health Assessment Questionnaire, *PASI* Psoriasis Area and Severity Index, *BMI* body mass index, *csDMARDs* conventional synthetic disease-modifying anti-rheumatic drugs, *TNFα* tumor necrosis factor α

The anti-TNFα was the bDMARD used in the majority of patients (*n* = 226, 85.61%), followed by ustekinumab (*n* = 23, 8.71%) and secukinumab (*n* = 15, 5.68%). In the period of observation, there were no patients undergoing ixekizumab, guselkumab, apremilast, abatacept, and tofacitinib.

Of the 117 switchers, 45 (38.46%) were initially on etanercept, 38 (32.48%) on adalimumab, 19 (16.24%) on infliximab, 8 (6.84%) on ustekinumab, 2 (1.71%) on golimumab, 2 (1.71%) on certolizumab, and 3 (2.56%) on secukinumab. Of the 147 non-switchers, 60 (40.82%) were initially on adalimumab, 48 (32.65%) on etanercept, 15 (10.2%) on ustekinumab, 12 (8.16%) on secukinumab, 8 (5.44%) on infliximab, 3 (2.04%) on golimumab, and 1 (0.68%) on certolizumab.

Overall, the survival rate of first bDMARD was good both in patients treated with anti-TNFα and in those undergoing anti-IL12/23 or anti-IL17 (75% at 2 years and 60% at 5 years). The group of patients undergoing anti-TNFα achieved a survival rate of about 50% after 10 years of treatment. We compared the survival rate for the main 3 anti-TNFα drugs administered (adalimumab, etanercept, and infliximab): the former 2 showed a higher probability of survival rate over 50% even after 10 years of treatment, whereas the survival rate of the former as first biologic was slightly below 50% after 10 years.

**Fig. 1:**
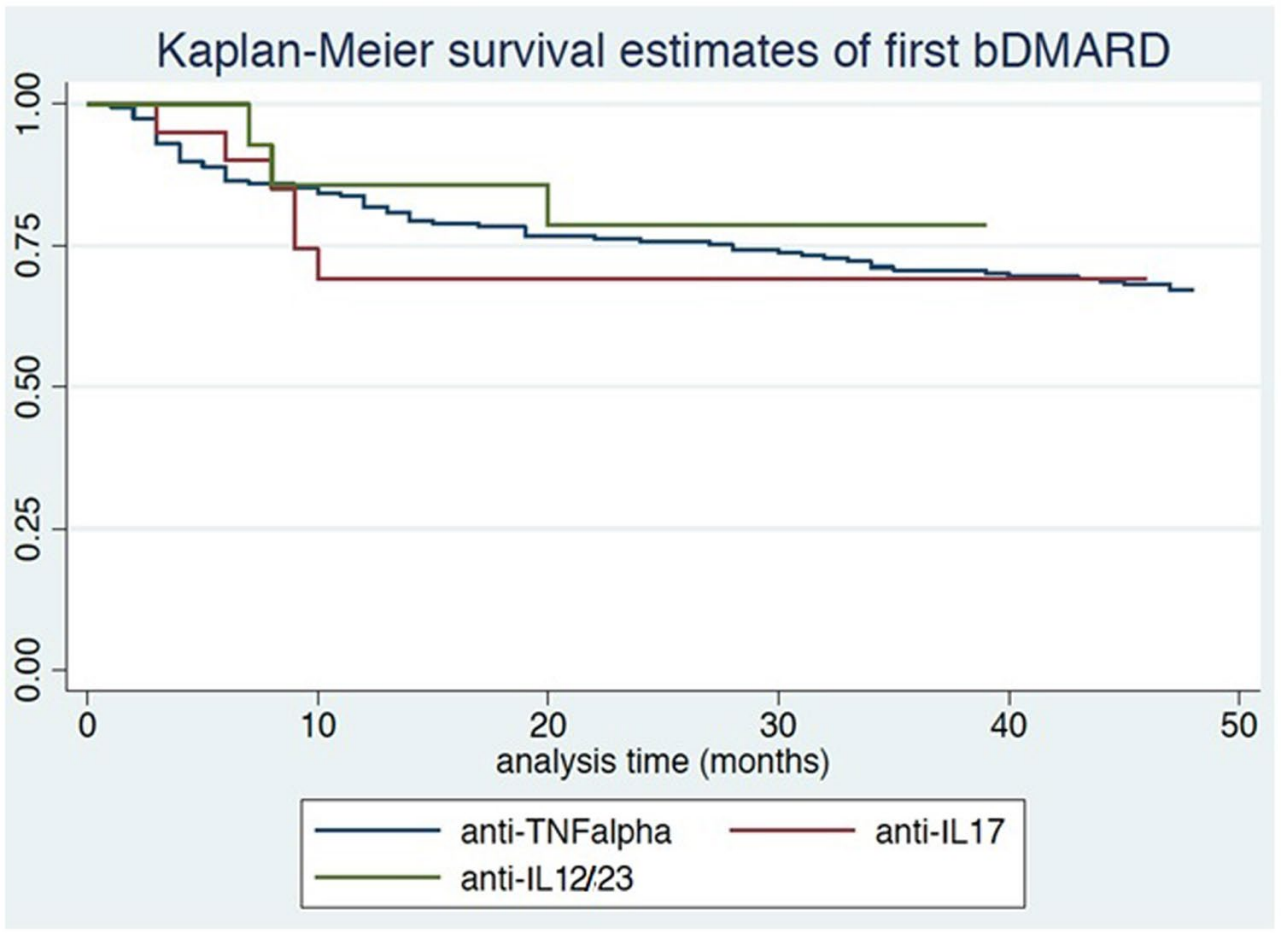
Kaplan–Meier survival curves of first bDMARD according to its mechanism of action

Survival curves for anti-TNFα (ustekinumab and secukinumab) did not diverge significantly (log-rank test = 0.83; *p* = 0.66; Fig. 1). There was no difference, in terms of survival for the second biologic, between patients who swapped and those who switched (log-rank test = 2.10, *p* = 0.147). The same was observed with Cox regression even after adjusting for the reason behind the therapeutic change (data not shown). We found no differences among all anti-TNFα biologics (infliximab, etanercept, adalimumab, certolizumab pegol, and golimumab) in terms of first or multiple bDMARD discontinuation (data not shown).

The principal reasons for bDMARD discontinuation were drug inefficacy (79 patients, 67.52%) and adverse events (38 patients, 25.7%). In more detail, these included lack of efficacy on arthritis (25 patients, 21.37%), lack of efficacy on cutaneous psoriasis (6 patients, 5.13%), loss of efficacy on arthritis (38 patients, 32.48%), loss of efficacy on cutaneous psoriasis (10 patients, 8.55%), recurrent or serious infections (10 patients; 8.55%), infusion reactions (14 patients, 11.97%), new onset of neoplasia (7 patients, 5.98%), severe comorbidities (2 patients, 1.71%), and biohumoral blood alterations (5 patients, 4.27%). No patient discontinued therapy due to non-compliance or pregnancy. In the comparison between switchers due to drug inefficacy and switchers due to adverse events, we observed a higher prevalence of psoriasis (*p* = 0.041) and dactylitis (*p* = 0.043) and a higher PASI (*p* = 0.045) in the former and a higher prevalence of comorbidities (*p* = 0.046) in the latter (Table 2). A detailed description of the principal reasons for discontinuation of each anti-TNFα biologic, anti-IL12/23, and anti-IL17 was reported in Supplementary Table 1.

**Table 2:** Baseline characteristics of switchers at the start of bDMARD therapy, according to the reason for subsequent switching

Characteristics	Inefficacy (*N* = 79)	Adverse event (*N* = 38)	*p* value
Female sex	43 (54.43%)	26 (68.42%)	0.65
Age (years)	58.0 (49.0–64.0)	56.0 (48.0–66.0)	0.07
Psoriatic arthritis duration (years)	15.0 (10.0–22.0)	16 (10.0–20.0)	0.91
Polyarticular arthritis	23 (29.11%)	9 (23.68%)	0.68
Mono/oligoarticular arthritis	36 (45.57%)	18 (47.37%)	0.86
Axial involvement	34 (43.08%)	14 (36.84%)	0.91
Psoriasis duration (years)	21.0 (14.0–30.0)	24.0 (11.0–33.0)	0.82
Family history	7 (8.86%)	7 (18.42%)	0.06
Psoriasis	**75 (94.93%)**	**27 (71.05%)**	**0.041**
Inflammatory bowel disease	1 (1.27%)	2 (5.26%)	0.16
Uveitis	0 (0%)	0 (0%)	0.10
Tender joints (66/68 joint count)	4.0 (2.0–7.0)	5.0 (2.0–9.0)	0.34
Swollen joints (66/68 joint count)	1.0 (0.0–3.0)	2.0 (0.0–5.0)	0.20
VAS pain 0–10	7.0 (5.0–8.0)	7.0 (5.0–7.5)	0.88
VAS global health 0–10	6.5 (4.5–8.0)	6.5 (5.1–7.0)	0.45
CRP (mg/L)	5.0 (3.0–11.5)	7.0 (3.0–12)	0.07
ESR (mm/h)	20.0 (10.0–30.0)	16.0 (10.0–29.0)	0.13
DAPSA	19.0 (14.0–27.0)	22.0 (18.0–26.0)	0.53
Leeds Enthesitis Index (0–6)	0.0 (0.0–1.0)	0.0 (0.0–0.0)	0.75
Dactylitis (presence/absence)	**13 (16.46%)**	**2 (5.26%)**	**0.043**
HAQ	1.0 (0.0–1.0)	1 (0.0–2.0)	0.061
PASI 0–72	**1.0 (0.0–3.0)**	**0 (0.0–1.0)**	**0.045**
Smoking			
Non-smokers	47 (59.49%)	32 (84.21%)	0.055
Ever smokers	32 (40.51%)	6 (15.79%)	
BMI	26 (24–284)	25.0 (22.0–27.0)	0.76
Updated Charlson Comorbidity Index	**1 (1–1)**	**1 (1–3)**	**0.046**
Association therapy with a csDMARDs	26 (32.91%)	13 (34.21%)	0.62
First-line biological drug			
Anti-TNFα	71 (89.87%)	35 (92.11%)	0.61
Ustekinumab	7 (88.60%)	1 (2.63%)	0.55
Secukinumab	1 (1.27%)	2 (5.26%)	0.92

Significant results are highlighted in bold. Categorical variables are shown as number (%). Continuous variables are shown as medians and interquartile range. *p* ≤ 0.05 (between inefficacy vs. adverse event)

*VAS* visual analogue scale, *CRP* C-reactive protein, *ESR* erythrocyte sedimentation rate, *DAPSA* Disease Activity in PSoriatic Arthritis, *HAQ* Health Assessment Questionnaire, *PASI* Psoriasis Area and Severity Index, *BMI* body mass index, *csDMARDs* conventional synthetic disease-modifying anti-rheumatic drugs, *TNFα* tumor necrosis factor α

The whole population achieved a significant decrease in tender/swollen joint count, VAS pain, VAS global health, PASI, LEI, HAQ, ESR, CRP, and DAPSA during a 15-year follow-up, with a trend of greater reduction of disease activity and improvement of functional index values in non-switchers vs. switchers (Table 3).

**Table 3:** Clinical, disease activity, and serological parameters of non-switchers (*n* = 147) and switchers (*n* = 117) among PsA patients during 15 years of follow-up (FU)

	Years to follow-up																*p*
	0	1	2	3	4	5	6	7	8	9	10	11	12	13	14	15	
	No. of patients																
	264	253	221	210	180	156	129	103	98	79	67	55	46	28	13	10	
SJ																	
Non-switchers	**2 (0–4)**	**0 (0–0)**	**0 (0–0)**	**0 (0–0)**	**0 (0–0)**	**0 (0–0)**	**0 (0–0)**	**0 (0–0)**	**0 (0–0)**	**0 (0–0)**	**0 (0–0)**	**0 (0–0)**	**0 (0–0)**	**0 (0–0)**	**0 (0–0)**	**0 (0–0)**	** < 0.01**
Switchers	**2.5 (0–6)**	**0 (0–2)**	**0 (0–1)**	**0 (0–1)**	**0 (0–0)**	**0 (0–0)**	**0 (0–0)**	**0 (0–0)**	**0 (0–0)**	**0 (0–0)**	**0 (0–0)**	**0 (0–2)**	**0 (0–2)**	**0 (0–2)**	**0 (0–2.5)**	**0 (0–0)**	** < 0.01**
TJ																	
Non-switchers	**5 (2–8)**	**1 (0–3)**	**0 (0–2)**	**0 (0–2)**	**0 (0–2)**	**0 (0–1)**	**0 (0–1)**	**0 (0–1)**	**0 (0–1)**	**0 (0–2)**	**0 (0–1)**	**0 (0–1)**	**0 (0–1)**	**0 (0–1)**	**0 (0–1)**	**0 (0–0)**	** < 0.001**
Switchers	**4 (2–8)**	**2 (1–6)**	**2 (0–5)**	**1 (0–4)**	**1 (0–4)**	**1 (0–3)**	**1 (0–2)**	**0 (0–2)**	**1 (0–2.3)**	**0 (0–2.25)**	**2 (0–3.5)**	**2.5 (0–5.3)**	**2 (0–8)**	**1 (0–3)**	**2 (1.8–6.3)**	**0 (0–2)**	** < 0.001**
VAS pain																	
Non-switchers	**7 (5.3–8)**	**3 (2–5)**	**3 (1.5–4)**	**3 (1.8–4.5)**	**3 (1.5–4.5)**	**3 (1.5–4.5)**	**2 (1.0–3.7)**	**2.8 (1.0–4.0)**	**2.4 (1.0–4.0)**	**2.0 (1.0–3.0)**	**2.0 (1.0–3.0)**	**3.0 (2.0–3.2)**	**2.5 (1.5–3.0)**	**2.0 (2.0–3.0)**	**3.0 (2.0–4.0)**	**2 (1.8–4.0)**	** < 0.001**
Switchers	**7.0 (5.0–7.5)**	**5.0 (3.0–7.0)**	**5.0 (3.0–7.0)**	**4.3 (2.5–7.0)**	**4.0 (2.7–6.3)**	**3.5 (2.0–6.0)**	**3.5 (2.5–5.0)**	**3.0 (2.5–5.0)**	**4.0 (2.9–5.7)**	**3.5 (2.0–5.6)**	**4.0 (3.0–6.6)**	**4.0 (2.0–6.1)**	**5.0 (3.0–7.0)**	**4.0 (1.0–6.0)**	**5.5 (3.8–7.3)**	**3.0 (2.0–5.0)**	** < 0.001**
VAS global health																	
Non-switchers	**6.0 (4.5–7.0)**	**29.5 (1.5–4.5)**	**25 (15–38.5)**	**2.0 (1.0–4.0)**	**2.5 (1.2–4.0)**	**2.5 (1.5–4.0)**	**2.0 (1.0–4.0)**	**2.1 (1.0–3.9)**	**2.0 (1.0–3.0)**	**2.0 (1.0–3.0)**	**2.2 (1.0–3.0)**	**2.1 (1.5–3.0)**	**2.0 (1.0–3.0)**	**2.0 (2.0–3.0)**	**3.0 (2.0–3.9)**	**2.5 (2.0–3.3)**	** < 0.001**
Switchers	**6.5 (5.0–8.0)**	**4.5 (28.8–6.5)**	**4.0 (2.5–6.5)**	**4.0 (2.5–6.0)**	**3.5 (2.0–5.6)**	**3.0 (2.0–5.5)**	**3.0 (1.9–5.6)**	**3.0 (1.5–4.5)**	**3.0 (2.1–5.3)**	**3.25 (2.0–4.85)**	**4.0 (2.7–6.3)**	**3.5 (2.5–5.0)**	**4.0 (2.0–5.5)**	**3.0 (2.2–5.0)**	**4.0 (2.8–6.0)**	**2.5 (1.5–3.0)**	** < 0.001**
LEI																	
Non-switchers	**0 (0–1)**	**0 (0–0)**	**0 (0–0)**	**0 (0–0)**	**0 (0–0)**	**0 (0–0)**	**0 (0–0)**	**0 (0–0)**	**0 (0–0)**	**0 (0–0)**	**0 (0–0)**	**0 (0–0)**	**0 (0–0)**	**0 (0–0)**	**0 (0–0)**	**0 (0–0)**	** < 0.05**
Switchers	**0 (0–1)**	**0 (0–0)**	**0 (0–0)**	**0 (0–0)**	**0 (0–0)**	**0 (0–0)**	**0 (0–0)**	**0 (0–0)**	**0 (0–0)**	**0 (0–0)**	**0 (0–0)**	**0 (0–0)**	**0 (0–0)**	**0 (0–0)**	**0 (0–0)**	**0 (0–0)**	** < 0.05**
PASI																	
Non-switchers	**1.0 (0.3–3.0)**	**0 (0.0–1.0)**	**0 (0.0–1.0)**	**0 (0.0–1.0)**	**0.2 (0.0–1.0)**	**0 (0.0–1.0)**	**0 (0.0–1.0)**	**0 (0.0–1.0)**	**0.3 (0.0–1.0)**	**0.20 (0.0–1.0)**	**0 (0.0–0.8)**	**0 (0.0–0.8)**	**0 (0.0–0.8)**	**0.5 (0.0–0.8)**	**0.2 (0.0–1.0)**	**0 (0.0–0.3)**	** < 0.001**
Switchers	**1.0 (0.0–2.7)**	**0 (0.0–1.2)**	**0 (0–1)**	**0 (0–1)**	**0 (0–1.4)**	**0 (0–1)**	**0 (0–1)**	**0 (0–1)**	**0 (0–1)**	**0.1 (0.0–1.5)**	**0.1 (0.0–2.0)**	**0 (0.0–0.9)**	**0 (0–1)**	**0 (0–0.5)**	**0 (0–0.5)**	**0 (0–0.4)**	** < 0.001**
ESR																	
Non-switchers	**17 (8–34)**	**8 (5–15.8)**	**9 (5–19)**	**7 (5–16.3)**	**10 (7–15)**	**10 (6–16.5)**	**10 (7–15)**	**10 (8–14)**	**10 (8–15)**	**10 (8–15)**	**10 (10–15)**	**10 (7–12)**	**10 (5–18.8)**	**7 (4–15.5)**	**7 (4–9.8)**	**6.5 (4–10.8)**	** < 0.001**
Switchers	**19 (9–36)**	**11 (9–20)**	**12 (7–20)**	**10 (8–20)**	**10 (7–21.5)**	**10 (8–23)**	**10 (7–16)**	**10 (7–19.8)**	**10 (7–23.3)**	**10 (8–17.75)**	**10 (7.5–31.5)**	**10 (8–20.8)**	**10 (7.5–20)**	**10 (5.5–21)**	**10 (4–21)**	**10 (7.8–23.8)**	** < 0.05**
PCR																	
Non-switchers	**6 (2.9–15.5)**	**3.05 (2–5.1)**	**3 (1.9–5.3)**	**2.9 (1.5–5.0)**	**2.9 (2.0–5.0)**	**2.9 (1.1–5.0)**	**3.7 (2.0–5.0)**	**3.0 (2.0–5.0)**	**3.5 (2.3–5.0)**	**3.0 (2.15.5.0)**	**2.9 (2.0–5.0)**	**2.9 (2.0–5.0)**	**2.9 (1.4–4.5)**	**2.9 (0.9–2.9)**	**2.9 (1.0–3.5)**	**1.3 (0.9–5.6)**	** < 0.001**
Switchers	**5.5 (2.9–12.0)**	**4.0 (2.9–6.0)**	**4.0 (2.9–6.0)**	**3.0 (2.9–6.0)**	**3.0 (2.4–5.0)**	**3.0 (2.2–5.0)**	**2.9 (2.0–4.9)**	**2.9 (2.1–5.0)**	**3.0 (2.9–5.1)**	**5.0 (2.9–6.0)**	**3.4 (2.9–5.0)**	**3.3 (2.2–7.9)**	**2.9 (1.5–5.0)**	**3.7 (2.2–7.0)**	**3.0 (1.3–5.3)**	**3.7 (3.3–7.9)**	** < 0.05**
HAQ																	
Non-switchers	**0.5 (0.3–1.0)**	**0.5 (0.3–0.8)**	**0.3 (0.3–0.6)**	**0.3 (0.3–0.6)**	**0.3 (0.3–0.6)**	**0.3 (0.3–0.6)**	**0.3 (0.3–0.6)**	**0.3 (0.3–0.3)**	**0.3 (0.3–0.4)**	**0.25 (0.25–0.25)**	**0.3 (0.3–0.3)**	**0.3 (0.3–0.3)**	**0.3 (0.3–0.5)**	**0.3 (0.3–0.3)**	**0.3 (0.3–0.3)**	**0.3 (0.3–0.3)**	** < 0.01**
Switchers	**0.62 (0.3–1.5)**	**0.6 (0.3–1.4)**	**0.7 (0.3–1.0)**	**0.6 (0.3–0.8)**	**0.6 (0.3–0.8)**	**0.5 (0.3–0.6)**	**0.3 (0.3–0.6)**	**0.5 (0.3–0.6)**	**0.4 (0.3–0.6)**	**0.63 (0.25–0.63)**	**0.5 (0.3–0.6)**	**0.5 (0.3–0.6)**	**0.5 (0.3–0.6)**	**0.3 (0.3–0.6)**	**0.3 (0.3–0.6)**	**0.3 (0.3–0.4)**	** < 0.01**
DAPSA																	
Non-switchers	**20.2 (15.1–27.9)**	**8 (5.2–12.3)**	**6.8 (3.8–10.6)**	**7.0 (4.3–10.7)**	**6.9 (3.9–11.2)**	**6.3 (4.0–9.7)**	**6.1 (3.2–8.4)**	**6.1 (2.9–8.8)**	**5.5 (2.8–9.7)**	**5.7 (3.34–8.22)**	**5.5 (2.0–8.0)**	**6.1 (4.2–8.0)**	**5.7 (3.5–7.6)**	**5.1 (4.1–7.3)**	**5.9 (4.3–10.6)**	**5.6 (4.1–8.9)**	** < 0.001**
Switchers	**18.9 (15.3–25.7)**	**13.7 (8.3–20.2)**	**12.4 (7.4–19.8)**	**11.1 (6.1–19.4)**	**10.1 (6.1–16.2)**	**8.6 (5.4–14.9)**	**8.2 (5.9–13.4)**	**7.8 (5.4–12.9)**	**9.5 (6.9–13.8)**	**8.12 (4.45–14.18)**	**10.2 (7.3–16.4)**	**12.5 (5.9–19.7)**	**12.2 (6.1–21.5)**	**10.4 (4.1–16.6)**	**12.9 (9.8–25.6)**	**7.1 (4.1–10.0)**	** < 0.001**

Significant results are highlighted in bold. Continuous variables are shown as medians and interquartile range. Values were computed by means of a chi-square test (for proportion) or the Mann–Whitney *U* test (for continuous data); *p* ≤ 0.05 T15 vs. T0

*VAS* visual analogue scale, *ESR* erythrocyte sedimentation rate, *DAPSA* Disease Activity in PSoriatic Arthritis, *HAQ* Health Assessment Questionnaire, *PASI* Psoriasis Area and Severity Index, *LEI* Leeds Enthesitis Index

The Cox PH model showed that female sex was independently associated with a higher risk of first bDMARD discontinuation (HR = 2.39; 95% CI: 1.50–3.81), while initiating therapy before 2015 was protective (HR = 0.40; 95% CI: 0.22–0.73). Other independent variables, including mechanism of action (HR = 0.76; 95% CI: 0.30–1.74 for secukinumab; HR = 0.53; 95% CI 0.15–1.86 for ustekinumab; reference: anti-TNFα), age (HR = 1.00; 95% CI: 0.99–1.03), baseline DAPSA (HR = 0.98; 95% CI: 0.96–1.00), PASI (HR = 0.95; 95% CI: 0.86–1.04), HAQ (HR = 1.29; 95% CI: 0.91–1.83), BMI (HR = 1.02; 95% CI: 0.98–1.07), polyarticular arthritis (HR = 1.23; 95% CI: 0.94–1.52), and comorbidities (HR = 1.10; 95% CI: 0.92–1.31), were not associated with the outcome “multi-failure” (Table 4). In the logistic regression model, only female sex was significantly associated with failure of multiple bDMARDs (OR = 1.99, 95% CI: 1.07–3.69) whereas bDMARD mechanism of action, age, and treatment initiation before 2015 were not independently associated with the outcome (Table 5).

**Table 4:** Cox regression model with first bDMARD discontinuation as outcome

Independent variables	First bDMARD discontinuation	
	Multivariable analysis	
	OR (95% CI)	*p*
Anti-IL17 as first drug	0.76 (0.29, 1.94)	0.567
Anti-IL12/23 as first drug	0.53 (0.15, 1.86)	0.325
Female sex	2.38 (1.49, 3.81)	** < 0.001**
Age	1.01 (0.99, 1.03)	0.322
BMI	1.02 (0.98, 1.07)	0.329
PASI baseline	0.95 (0.86, 1.04)	0.263
DAPSA baseline	0.98 (0.96, 1.01)	0.153
HAQ baseline	1.29 (0.91, 1.83)	0.155
Charlson Comorbidity Index	1.09 (0.92, 1.31)	0.299
Polyarticular arthritis	1.23 (0.94, 1.52)	0.151
bDMARD initiation < 2015	0.41 (0.22, 0.73)	**0.003**

Significant results are highlighted in bold. *p* ≤ 0.05

*bDMARD* biological disease-modifying anti-rheumatic drug, *IL* interleukin, *BMI* body mass index, *PASI* Psoriasis Area and Severity Index, *HAQ* Health Assessment Questionnaire, *DAPSA* Disease Activity Index for Psoriatic Arthritis, *CI* confidence interval

**Table 5:** Multivariable logistic regression model with failure of multiple (≥ 2) bDMARD therapies as outcome

Independent variables	Failure of multiple (≥ 2) bDMARD therapies	
	Multivariable analysis	
	OR (95% CI)	*p*
Anti-IL17 as first drug	− 1.91 (− 4.06, 0.24)	0.082
Anti-IL12/23 as first drug	− 0.64 (− 2.31, 1.02)	0.447
Female sex	0.69 (0.07, 1.31)	**0.030**
Age	0.02 (0.01, 0.04)	0.229
bDMARD initiation < 2015	0.09 (0.72, 0.91)	0.060

Significant result is highlighted in bold. *p* ≤ 0.05

*bDMARD* biological disease-modifying anti-rheumatic drug, *IL* interleukin, *CI* confidence interval

## Discussion

Our study evaluated 264 PsA patients who had been undergoing biologics for a maximum during 2004–2020. Clinical and demographic characteristics of our study population were in line with the main nationwide registries BIOBADASER, BSRBR, DANBIO, and NOR-DMARD [26–30]. One hundred and seventeen (44.32%) PsA patients treated with a first bDMARD (anti-TNFα, ustekinumab or secukinumab) switched to another. Loss of efficacy, lack of efficacy, and adverse events were the main reasons for switching to another anti-TNFα or swapping for another biologic altogether [34–37]. Notably, the main reason for switching in our study was drug inefficacy (67.52%). The different mechanisms of action (anti-TNFα, anti-IL12/23, anti-IL17) were not associated with a higher probability of switching. Observational studies have reported a sustained clinical response at 5 years, with satisfactory infliximab and adalimumab survival rates and higher etanercept survival [12]. Survival rate of patients receiving anti-TNFα treatment appears to be greater in PsA vs. rheumatoid arthritis (RA) patients [26, 38, 39]. In our patients, the survival rate of the first bDMARD was 60% at 5 years and 75% at 2 years both in patients treated with anti-TNFα and in those undergoing anti-IL12/23 or anti-IL17. A survival rate > 50% at 10 years was observed among patients undergoing anti-TNFα. The persistence of treatment with first bDMARD was in line with the current literature [12, 38, 39] whereas the percentage of switchers (44.32%) was higher vs. nationwide registries, in which 20–35% of SpA patients had switched first TNFα inhibitors, though in those latter studies, the duration of follow-up was shorter (mean 1–5 years vs. 2–15 years). Overall, drug survival rate of the second anti-TNFα appears to be lower vs. the first anti-TNFα [40]. In the BIOBADASER registry, among 4706 patients with chronic arthritis—including RA, PsA, and ankylosing spondylitis (AS)—10% had been treated with more than one anti-TNFα over a 4-year period [41]; 88% of PsA patients continued with their first anti-TNFα drug for 12 months vs. 83% of RA patients [26]. Recently, a Norwegian study found that 77.3% of PsA vs. 65.4% of RA patients continued with their first anti-TNFα for 12 months [42]. A BSRBR observational study found that 31% of 566 PsA patients switched treatment and were followed up for a mean duration of 2.3 years [43]. Similarly, a French single-center study found that 64% of rheumatic patients continued their first anti-TNFα treatment for 12 months [38]. Conversely, the DANBIO registry reported that 39% of 1422 patients, initiated anti-TNFα, then switched to a second biologic over a 10-year follow-up period, as corroborated by our findings [29]. However, nationwide registries provide scarce data and few observational studies have investigated anti-TNFα switching in patients with PsA (e.g., 5-year estimated drug survival for first-time switchers was 51% in a Southern Sweden cohort study) [44]. A real-life French study reported switching rates of 26–32% in patients with SpA [45]. Overall, drug survival rate of the second anti-TNFα appears to be lower vs. that of the first anti-TNFα [40, 46]. As previously reported in the literature, we also found a decrease of disease activity parameters in our patients in treatment with bDMARDs with a stronger trend in non-switchers [26–30]. We observed no differences as regards survival rate to the second biological drug, between patients who swapped/switched biological therapy, a finding in line with EULAR recommendations for therapeutic switching and evidence on efficacy [9]. Moreover, we were able to confirm previous reports of a low incidence of serious and/or recurrent infections—one of the most feared causes of drug withdrawal [47]. In fact, only 8.55% of our patients required a switch due to recurrent infections. Behrens et al. [48] underlined that available evidence on the efficacy and safety of anti-TNFα monotherapy vs. add-on methotrexate therapy showed little or no improvement with combination therapy, though the use of concomitant methotrexate appears to prolong anti-TNFα drug survival by reducing the development of TNFα inhibitor antibodies. Likewise, the percentage of patients treated with combined therapy in our study (bDMARDs + csDMARDs) was similar in non-switchers (38.10%) and in switchers (33.33%). Therefore, concurrent csDMARD use, and specifically methotrexate, did not yield better response vs. monotherapy.

Our study investigated potential predictors of switching in first- and second-line biologics.

To the best of our knowledge, few studies have formally explored possible predictors of drug discontinuation in patients with SpA and PsA treated with anti-TNFα [43]. Kristensen et al. [18] suggested that concomitant use of methotrexate and elevated C-reactive protein levels was associated with treatment continuation using anti-TNFα drugs. Gomez-Reino et al. [41] reported that older age was a predictor of drug discontinuation, while Heiberg et al. [42] found that higher baseline disease activity and female sex were associated with treatment continuation.

We were able to corroborate previous reports in the literature pertaining to a higher discontinuation rate of first- and second-line biologics among females [37, 49, 50]. In fact, Iannone et al. [51] reported that male PsA patients showed a 50% risk of discontinuation and were 60% more likely to achieve long-term stable minimal disease activity. Female PsA patients more frequently present a polyarticular pattern, often compounded by fibromyalgia, which amplifies the perception of pain and fatigue and therefore negatively impacts self-reported assessment of disease activity [52]. Furthermore, concomitant fibromyalgia may constitute a challenge to the therapeutic strategy in female PsA patients, as fibromyalgia-associated symptoms can alter the assessment of clinical response to treatments over time. We also observed that switchers more frequently had polyarticular arthritis, probably due to a more severe disease, without differences related to reason for switching (inefficacy or adverse event), although this subtype did not appear to be a significantly negative prognostic factor for time-to-first bDMARD discontinuation. These findings corroborate a previous report by literature [49]. Conversely, the mono-oligoarticular subtype and axial disease did not appear to influence treatment response and drug discontinuation. We also found a trend towards better drug survival in patients who initiated bDMARDs before 2015, probably reflecting the limited number of available therapies with specific mechanism of action. We hypothesized that another possible reason may be that before 2015, patients were usually switched to biological drugs following multiple csDMARD failure and after a longer disease course. Thus, even a partial response could be deemed successful and physicians would maintain the same treatment longer. The presence of comorbidities was higher among switchers though it was not defined as a predictor of higher withdrawal rates. Although current smoking was also found to be an independent predictor of discontinuation of biological therapies [40], we were not able to confirm this association despite about 34% of our patients being current or previous smokers. Interestingly, we observed a high prevalence of psoriasis and higher PASI at the time of bDMARD initiation among switchers and in cases where bDMARD discontinuation was due to a lack of efficacy.

The strengths of our study were the evaluation of predictors of switching of bDMARDs and the collection of data about efficacy and reasons for switching in a real-life setting. Some of our limitations were the retrospective nature of the study design; the choice of first bDMARD influenced by factors such as the treating physician’s preference and the timing of the availability of the novel mechanism of action; the severity bias of enrolled PsA patients, who attended a tertiary center and therefore could present a more difficult-to-treat and aggressive disease; and the small sample size that does not allow to evaluate fine differences between different anti-TNFα drugs and between anti-TNFα and other bDMARDs such as anti-IL17 and anti-IL12/23.

## Conclusions

The drug survival in PsA patients was greater for the first biologic administered, which could arise from a better drug selection tailored to each patient’s prevalent clinical manifestations and comorbidities according to the EULAR/GRAPPA/SIR recommendations.

Overall, almost half of PsA patients treated with a first bDMARDs switched to another during the 15 years of follow-up. At 2 years and 5 years, the survival rate of the first bDMARD was from really optimal to good in over 50% of the PsA patients, without a significant difference in patients undergoing anti-TNFα, anti-IL12/23, and anti-IL17 biological agents. In case of failure of the first bDMARD, switching/swapping proved to be a good treatment option, as reflected by a persistently satisfactory effectiveness with second-line bDMARDs and so subsequent switches. Discontinuation or switching of first bDMARD due to tolerability issues or infections occurred rarely in PsA with respect to RA.

Furthermore, female sex may constitute a predisposing risk factor for flare and therapeutic switches. Discontinuation or switching of biologics due to mechanism of action, comorbidities tolerability, and BMI did not seem to impact first bDMARD withdrawal. Lack of efficacy does not appear to be a frequent occurrence in PsA vs. other rheumatic diseases such as RA.

## Supplementary Information

Below is the link to the electronic supplementary material.

Supplementary file1 (DOCX 13 KB)
