# Supplementary material for: Predictive factors for switching in patients with psoriatic arthritis undergoing anti-TNFα, anti-IL12/23, or anti-IL17 drugs: a 15-year monocentric real-life study
Source: Clin Rheumatol. 2021 Jun 16;40(11):4569–80. doi: 10.1007/s10067-021-05799-0 (PMC8519923; doi:10.1007/s10067-021-05799-0)
Supplement: Supplementary file 1 — Supplementary file1 (DOCX 13 KB) [file 10067_2021_5799_MOESM1_ESM.docx]

**Supplementary Table 1.** **Baseline characteristics of switchers (for each bDMARD class) at the start of bDMARDs therapy, according to the reason for subsequent switching**.

| **Class of bDMARDs** | **total switchers** | **Inefficacy** | **Adverse event** | **p-value** |
| --- | --- | --- | --- | --- |
|  | **N=117** | **N=79** | **N=38** |  |
| Etanercept | 45 (38.46%) | 30 (37.97%) | 15 (39.47%) | 0.25 |
| Adalimumab | 38 (32.48%) | 28 (35.44%) | 10 (26.32%) | 0.08 |
| Infliximab | 19 (16.24%) | 11 (13.92%) | 8 (21.05%) | 0.44 |
| Golimumab | 2 (1.71%) | 1 (1.27%) | 1 (2.63%) | 0.52 |
| Certolizumab | 2 (1.71%) | 0 (0%) | 2 (5.26%) | 0.07 |
| Ustekinumab | 8 (6.84%) | 5 (6.33%) | 3 (7.89%) | 0.23 |
| Secukinumab | 3 (2.56%) | 2 (2.53%) | 1 (2.63%) | 0.31 |

**Legend**: Data shown as number (%). p≤0.05 (between inefficacy-group and adverse event-group). bDMARDs=biological Disease-Modifying Anti-Rheumatic Drugs.
